# Supplementary material for: The Statin Target Hmgcr Regulates Energy Metabolism and Food Intake through Central Mechanisms
Source: Cells. 2022 Mar 11;11(6):970. doi: 10.3390/cells11060970 (PMC8946516; doi:10.3390/cells11060970)
Supplement: Supplementary file 1 [file cells-11-00970-s001.zip › cells-1612659-supplementary.pdf]

Supplemental Figures

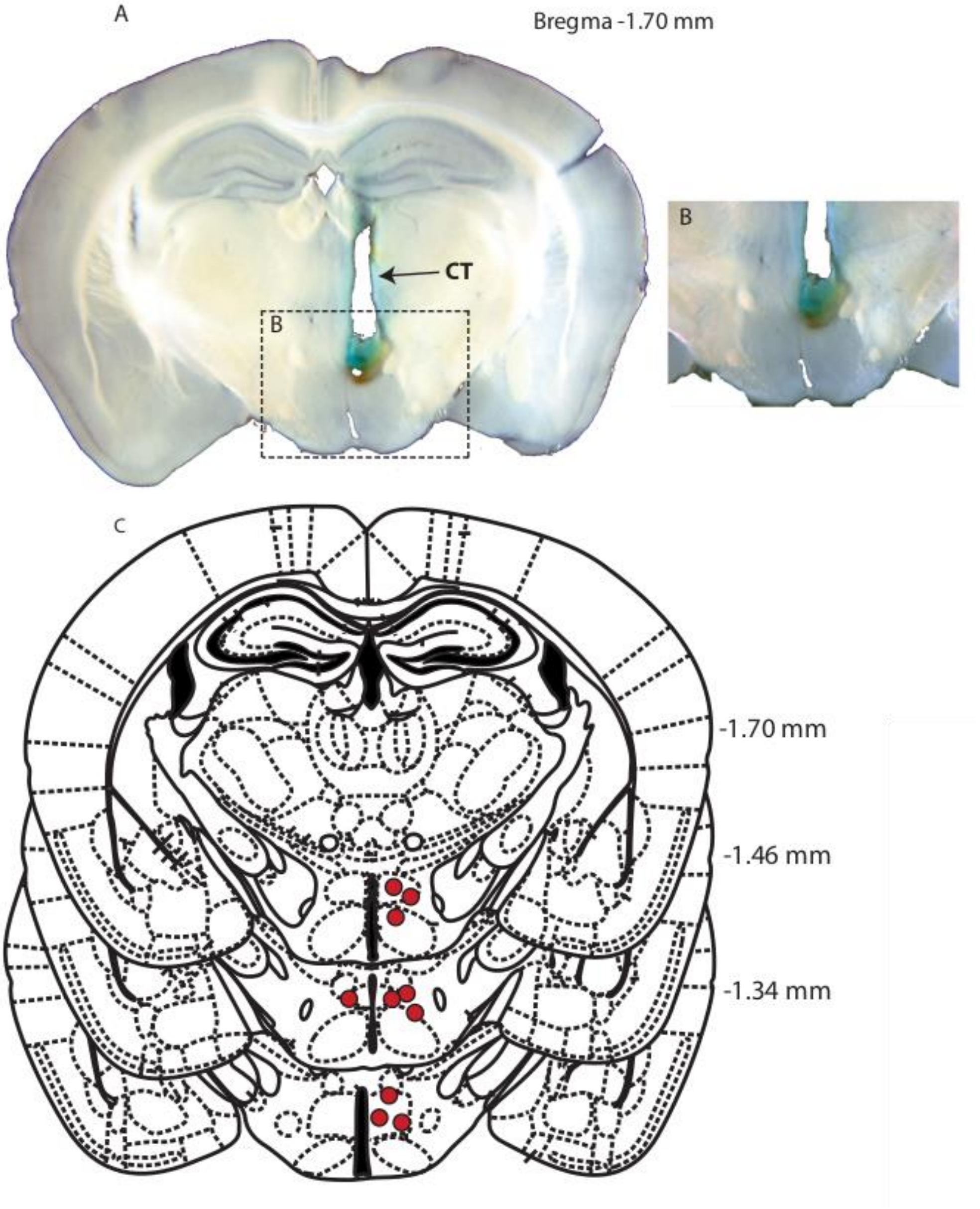

Supplemental Figure S1. **Verification of injection sites** (A) Mouse brain section showing where methyl blue was injected into guide cannula to confirm correct placement. (B) enlarged section from (B, square box), showing methyl blue around cannula insertion site. (C) Diagram showing cannula insertion sites from all mice used in the experiment.

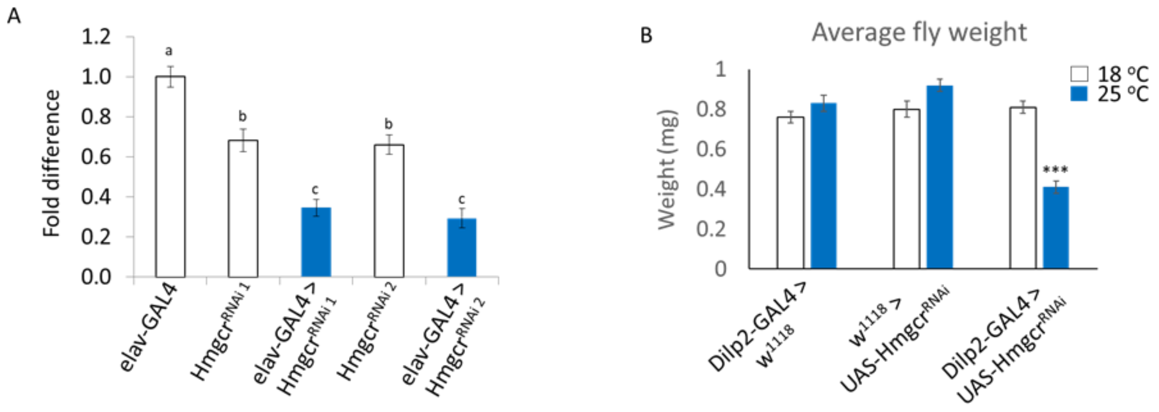

Supplemental Figure S2. **Loss of *Hmgcr* expression in Dilp2 cells reduces adult fly weight.** (A) Two different *Hmgcr UAS-RNAi* lines were crossed to the nervous system driver *elav-GAL4* and equally aged 5-7 day old adult males were used for qPCR analysis to determine if the RNAi lines were functional. (*UAS-Hmgcr*^{RNAi1} *= P{KK101807}sVIE-260B, UAS-Hmgcr*^{RNAi2} = *P{UAS-RNAi-HMGCR}*our10367-R3). (n = 10 replicates, consisting of 10 male whole bodies, 5-7 days old). Different letters indicate similar groups (i.e. 'a' is significantly different than 'b' or 'c' and so on, one-way ANOVA with Tukey's post hoc test for multiple comparisons was performed). Error bars = SEM. (B) *Hmgcr* was knocked down in the insulin-producing cells, the average body weight of adult male flies decreased in the experimental group (*Dilp2-Gal4>UAS-Hmgcr*^{RNAi}) compared to the controls (*Dilp2-Gal4>w*^{1118} and *w*^{1118}*>UAS-Hmgcr*^{RNAi}) when reared at 25 ^{o}C. When reared at 18 ^{o}C, there was no difference in the body weight in both experimental and control groups. (n=10 for each group, One-Way ANOVA with a Bonferroni posthoc test was performed to detect the significance. *** $P < 0.001$) Error bars = SEM.

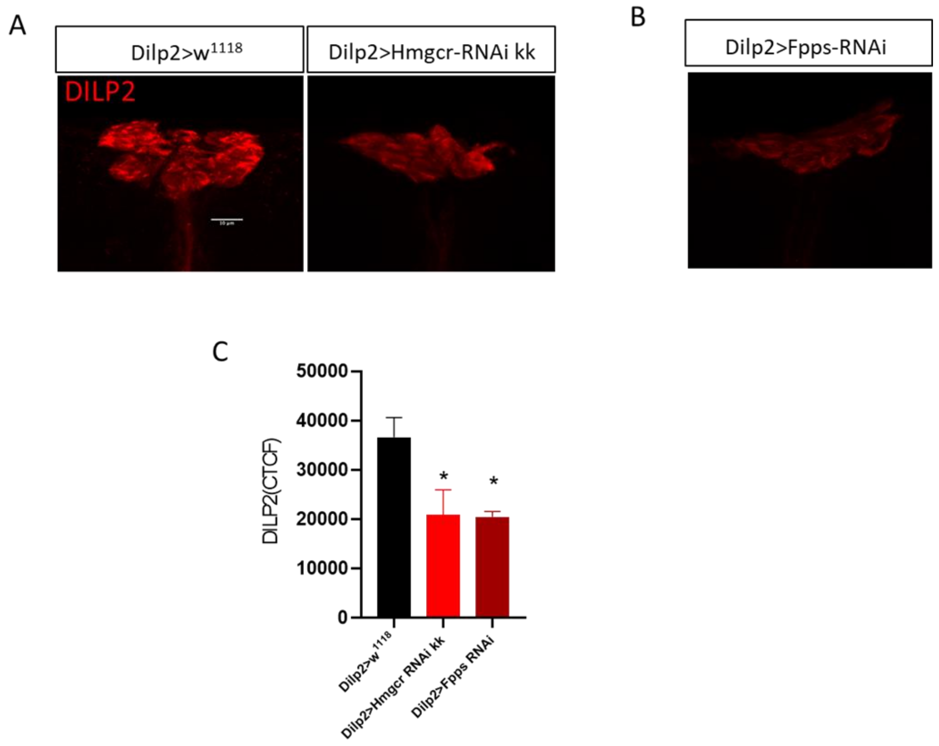

Supplemental Figure S3. **Loss of *Fpps* in Dilp2 cells reduces ILP2 expression**. Manipulating *Hmgcr* and the downstream of mevalonate pathway (*Fpps*) affected on ILP2 levels in the IPCs. (A-C) the ILP2 levels significantly decreased in both experimental groups (*Dilp2-GAL4>UAS Hmgcr* RNAi and *Dilp2-GAL4>Fpps* RNAi) compared to the equally aged control *Dilp2-GAL4>*w^{1118}. CTCF: corrected total cell fluorescence. (n = 10 male fly heads per genotype, 5-7 days old. One-way ANOVA with Tukey's post hoc test for multiple comparisons was performed, * $P < 0.05$. In the graph error bars = S.E.M.)
